# Cystatin C predicts renal function impairment after partial or radical tumor nephrectomy

**DOI:** 10.1007/s11255-021-02957-w

**Published:** 2021-07-16

**Authors:** Mike Wenzel, Hang Yu, Annemarie Uhlig, Christoph Würnschimmel, Manuel Wallbach, Andreas Becker, Margit Fisch, Felix K. H. Chun, Christian P. Meyer, Marianne Leitsmann

**Affiliations:** 1 Department of Urology, University Hospital Frankfurt, Goethe University Frankfurt, Frankfurt, Germany; 2 Cancer Prognostics and Health Outcomes Unit, Division of Urology, University of Montreal Health Center, Montreal, QC Canada; 3 Department of Urology, University Medical Center Hamburg-Eppendorf, Hamburg, Germany; 4 Department of Urology, University Medical Center Göttingen, Göttingen, Germany; 5 Martini-Klinik Prostate Cancer Center, University Hospital Hamburg-Eppendorf, Hamburg, Germany; 6 Department of Nephrology, University Medical Center Göttingen, Göttingen, Germany

**Keywords:** Cystatin C, Nephrectomy, Renal cell carcinoma, GFR, Acute kidney injury

## Abstract

**Purpose:**

To test the value of preoperative and postoperative cystatin C (CysC) as a predictor on kidney function after partial (PN) or radical nephrectomy (RN) in renal cell carcinoma (RCC) patients with normal preoperative renal function.

**Methods:**

From 01/2011 to 12/2014, 195 consecutive RCC patients with a preoperative estimated glomerular filtration rate (eGFR) > 60 ml/min/1.73m^2^ underwent surgical RCC treatment with either PN or RN. Logistic and linear regression models tested for the effect of CysC as a predictor of new-onset chronic kidney disease in follow-up (eGFR < 60 ml/min/1.73m^2^). Moreover, postoperative CysC and creatinine values were compared for kidney function estimation.

**Results:**

Of 195 patients, 129 (66.2%) underwent PN. In postoperative and in follow-up setting (median 14 months, IQR 10–20), rates of eGFR < 60 ml/min/1.73m^2^ were 55.9 and 30.2%. In multivariable logistic regression models, preoperative CysC [odds ratio (OR): 18.3] and RN (OR: 13.5) were independent predictors for a reduced eGFR < 60 ml/min/1.73m^2^ in follow-up (both *p* < 0.01), while creatinine was not. In multivariable linear regression models, a difference of the preoperative CysC level of 0.1 mg/dl estimated an eGFR decline in follow-up of about 5.8 ml/min/1.73m^2^. Finally, we observed a plateau of postoperative creatinine values in the range of 1.2–1.3 mg/dl, when graphically depicted vs. postoperative CysC values (‘creatinine blind area’).

**Conclusion:**

Preoperative CysC predicts renal function impairment following RCC surgery. Furthermore, CysC might be superior to creatinine for renal function monitoring in the early postoperative setting.

## Introduction

In localized renal cell carcinoma (RCC), surgical treatment with either nephron-sparing partial nephrectomy (PN) or radial nephrectomy (RN) is associated with excellent oncological outcomes [1–3]. However, all nephrectomy patients are at increased risk for renal function decline occurring as short-term acute kidney injury (AKI) or subsequent chronic kidney disease (CKD) [4, 5]. Within this context, RCC patients are at higher risk for postoperative new-onset cardiovascular morbidity and in the further mortality [6, 7]. In consequence, it is suggested to perform nephron-sparing surgery, whenever possible, as recommended in EAU guidelines [8]. In several studies, the estimated glomerular filtration rate (eGFR) or patients’ comorbidity profile was associated with postoperative AKI or long-term CKD after PN or RN [4–6, 9–12].

Moreover, multiple studies showed a better accuracy of cystatin C (CysC), relative to serum creatinine, to estimate kidney function through eGFR in surgically and non-surgically treated patients [13–15]. Several studies showed that CysC predicts short-term postoperative AKI and long-term kidney impairment [15–18]. Additionally, it was also found that creatinine, which is used in hospital standard routine, may over- and underestimate kidney function regarding patients’ age category [19]. In consequence, creatinine may not be the best parameter for kidney function monitoring after RN or PN and few if any is known about the role of CysC in surgically treated RCC patients regarding long-term kidney function. These observations make it necessary to investigate if CysC is potentially a better marker for risk stratification, especially in patients with mildly to moderately impaired kidney function and within the ‘creatinine blind area’, to counsel and classify patients even more accurate according to risk profiles and possibly postoperative monitoring [20].

We addressed this knowledge gap and hypothesized that CysC may play a crucial role in outcome measurements of kidney function after surgical treatment with RN or PN for RCC patients. Moreover, we postulated that CysC may be less influenced by patient’s age than creatinine.

## Methods

### Study population

The current study was performed in accordance with the Declaration of Helsinki and approved by the local ethic committee of the University Hamburg-Eppendorf (number PV4219). All consecutive patients who underwent surgical treatment with either PN or PN for RCC between 01/2011 and 12/2014 at the Department of Urology, University Medical Center Hamburg-Eppendorf, Germany, were retrospectively identified. Exclusion criteria consisted of patients with preoperative impairment of kidney function, defined as eGFR < 60 ml/min/1.73^2^ (*n* = 54), non-RCC postoperative histology (*n* = 17), surgical treatment for kidney trauma (*n* = 1) and death during follow-up (*n* = 9). These selection criteria resulted in 195 eligible surgically treated patients.

### Patient characteristics, renal function and AKI evaluation

From patient’s data files patient’s baseline characteristics according to blood, urine and tumor values/characteristics were collected. All RCC patients were surgically treated with either open, laparoscopic or robotic RN or PN. Preoperative and postoperative unsuspicious CysC values were defined as ≤ 0.96 mg/l, as previously described [21].

Provided data regarding eGFR, were calculated in accordance with the four variable MDRD Study Equation: GFR in ml/min/1.73^2^ = 186  ×  (S-Creatinine − 1.154)  ×  (age − 0.203) × 1.212 (if African American race/ethnicity) × 0.742 (if female sex) [22, 23]. Provided data regarding AKI were defined as either an ≥ 50% increase of the postoperative blood serum creatinine relative to preoperative blood serum creatinine. Alternatively, it was defined as an absolute increase of the postoperative blood serum creatinine of ≥ 0.3 mg/dl on first postoperative day after RN or PN in accordance with the current AKI criteria by KDIGO [24].

### Endpoints and follow-up

Primary endpoint of the study was the assessment of postoperative (day 1) and follow-up kidney function with regard to preoperative and postoperative CysC blood levels. Kidney function impairment in follow-up was defined as an eGFR < 60 ml/min/1.73^2^, in accordance with current definitions [25]. Patients underwent a systematic follow-up through a standardized survey supported by the treating urologist or family doctor. The survey consisted of questions addressing baseline characteristics [e.g., weight, body mass index (BMI) or blood pressure values], new-onset diseases and blood and urine values, if available. Secondary endpoints of the current study consisted of the relationship between first-day postoperative creatinine and CysC blood values in patients with non-elevated preoperative CysC.

### Statistical analyses

Descriptive statistics included frequencies and proportions for categorical and medians and interquartile ranges (IQR) for continuous variables. The Chi-square, the *t* test and Kruskal–Wallis tests assessed differences among groups as appropriate.

Uni- and multivariable logistic, as well as linear regression models were fitted to assess the value of CysC as a predictor of kidney function in follow-up after PN or RN. Significant variables in univariable logistic regression models, were included in multivariable models. Moreover, loess-plots were used to depict the correlation between first-day postoperative creatinine and postoperative CysC in surgically treated RCC patients. Subsequently, same tabulations were made for different age cut-offs and in postoperative AKI patients.

All statistical analyses were performed using the R statistical package system 3.4.3 (R Foundation for Statistical Computing, Vienna, Austria), with a two-sided significance level set at *p* < 0.05 [26].

## Results

### Descriptive baseline characteristics

Of all eligible 195 patients (Table 1), median age was 60 years and 134 were males (68.7%). Overall, 129 (66.2%) underwent PN vs. 66 (33.8%) RN. Open surgery was performed predominately (77.4%), followed by laparoscopic (15.9%) and robotic (6.7%) approaches. Preoperative creatinine, eGFR and CysC were respectively 0.9 mg/dl (IQR 0.8–1.0), 88 ml/min/1.73m^2^ (IQR 76–103) and 0.8 mg/l (IQR 0.7–0.9). In the entire cohort, rates of postoperative and follow-up eGFR < 60 ml/min/1.73m^2^ were respectively 55.9 and 30.2%. In particular, at follow-up, 75.9% of patients had CKD G3a (eGFR 45–59 ml/min/1.73m^2^/ and 24.1% of had CKD G3b (eGFR 30–44 ml/min/1.73m^2^).

**Table 1:** Descriptive characteristics of 195 patients who underwent either partial (PN) or radical nephrectomy (RN) at the University Hospital Hamburg-Eppendorf, stratified according to postoperative elevated (≥ 0.96 mg/dl) vs. normal (< 0.96 mg/dl) cystatin c level

Variable	Overall *n* = 195	Postoperative normal cystatin c *n* = 90 (46.2%)	Postoperative elevated cystatin c *n* = 105 (53.8%)	*p* value
Age, years				
Median (IQR)	60 (50–70)	59 (49–68)	61 (53–72)	0.042
Preoperative creatinine, mg/dl				
Median (IQR)	0.9 (0.8–1.0)	0.8 (0.7–0.9)	0.9 (0.8–1.1)	< 0.001
Preoperative eGFR, in ml/min/1.73m^2^				
Median (IQR)	88 (76–103)	95 (78–106)	83 (71–94)	< 0.001
Preoperative cystatin c, mg/dl				
Median (IQR)	0.8 (0.7–0.9)	0.7 (0.7–0.8)	0.8 (0.7–1.0)	< 0.001
Tumorsize, cm				
Median (IQR)	4.2 (2.4–5.8)	3.4 (2.1–4.7)	4.6 (3.0–6.5)	< 0.01
Postoperative creatinine, mg/dl				
Median (IQR)	1.2 (1.0–1.4)	1.1 (1.0–1.3)	1.3 (1.1–1.6)	< 0.001
Postoperative eGFR, in ml/min/1.73m^2^				
Median (IQR)	58 (48–71)	63 (54–78)	52 (42–63)	< 0.001
Postoperative cystatin c, mg/dl				
Median (IQR)	1.2 (1.0–1.5)	1.1 (1.0–1.3)	1.4 (1.1–1.7)	< 0.001
Follow-up eGFR, in ml/min/1.73m^2^				
Median (IQR)	67 (53–84)	69 (62–89)	63 (52–83)	0.3
Sex				
Female	61 (31.3)	33 (36.7)	28 (26.7)	0.2
Male	134 (68.7)	57 (63.3)	77 (73.3)	
BMI				
≥ 30	46 (23.6)	17 (18.9)	29 (27.6)	0.2
Type of surgery				
PN	129 (66.2)	75 (83.3)	54 (51.4)	0.001
RN	66 (33.8)	15 (16.7)	51 (48.6)	
Surgical approach				
Robotic	13 (6.7)	8 (8.9)	5 (4.8)	0.4
Laparoscopic	31 (15.9)	12 (13.3)	19 (18.1)	
Open	151 (77.4)	70 (77.8)	81 (77.1)	
Ischemia				
Yes	50 (25.6)	30 (33.3)	20 (19.0)	0.01
Preoperative hypertension				
Yes	75 (38.5)	35 (38.9)	40 (38.1)	0.7
Preoperative diabetes				
Yes	26 (13.3)	5 (5.6)	21 (20.0)	< 0.01
Preoperative proteinuria				
Yes	39 (20)	14 (15.6)	25 (23.8)	0.2
pT-stage				
T1	121 (62.0)	56 (62.2)	64 (61)	0.5
T2	17 (8.7)	5 (5.6)	13 (12.4)	
T3	11 (5.6)	4 (4.4)	7 (6.7)	
T4	1 (0.5)	0 (0)	1 (1)	
Postoperative proteinuria				
Yes	90 (46.2)	58 (64.4)	32 (30.5)	0.1
Postoperative acute kidney injury				
Yes	127 (65.1)	53 (58.9)	74 (70.5)	0.1

*IQR* interquartile range, *BMI* body mass index

Further stratification according to postoperative elevated or normal CysC level was made (Table 1). Here, 105 (53.8%) of all 195 patients had elevated CysC levels postoperatively. Of these, 74 (70.5%) and 72 (68.6%) exhibited postoperative AKI and eGFR < 60 ml/min/1.73m^2^. Moreover, patients with elevated postoperative CysC were older (61 vs. 59), exhibited higher rates of preoperative diabetes (20.0 vs. 5.6%, all *p* < 0.05) than patients with normal postoperative CysC. Additionally, patients with postoperative elevated CysC more frequently underwent RN (48.6 vs. 16.7%) and exhibited larger tumor sizes (4.6 vs. 3.4 cm).

### Cystatin C as a predictor for kidney function impairment at follow-up

Median follow-up duration was 14 months (IQR 10–20) and of all patients, 46.2% (*n* = 95) participated in the follow-up. At baseline, median eGFR was 88 vs. 67 ml/min/1.73m^2^ at follow-up (*p* = 0.2). In univariable logistic regression models (Table 2), preoperative CysC [odds ratio (OR): 9.9, confidence interval (CI): 3.2–24.9] and postoperative CysC (OR: 6.5, CI: 2.4–21.3, both *p* < 0.001) were associated with an eGFR < 60 ml/min/1.73m^2^. Moreover, tumor size (OR: 1.41, CI: 1.2–1.8), postoperative AKI (OR: 4.3, CI: 1.5–13.8) and RN (OR: 16.6, CI: 5.9–51.9) were associated with an eGFR < 60 ml/min/1.73m^2^ at follow-up (all *p* < 0.01).

**Table 2:** Univariable and multivariable logistic regression models predicting estimated glomerular filtration rate (eGFR) < 60 ml/min/1.73m^2^ in follow-up, after either partial (PN) or radical nephrectomy (RN) for renal cell carcinoma

	Univariable			Multivariable		
	OR	95% CI	*p* value	OR	95% CI	*p* value
Preoperative cystatin C	9.91	3.24–24.86	< 0.001	18.33	2.69–214.88	< 0.01
Postoperative cystatin C	6.51	2.36–21.27	< 0.001	1.32	0.23–7.77	0.7
Age at diagnosis	1.04	0.99–1.09	0.1	1.04	0.97–1.13	0.3
Tumor size	1.41	1.15–1.80	< 0.01	1.15	0.97–1.13	0.3
No postoperative AKI	1 (Ref.)	–	–	1	–	–
Postoperative AKI	4.25	1.54–13.84	< 0.01	3.35	0.57–26.85	0.2
PN	1 (Ref.)	–	–	1	–	–
RN	16.63	5.94–51.87	< 0.001	13.52	2.85–89.6	< 0.01
BMI	1.06	0.98–1.16	0.2	1.12	0.98–1.29	0.08
No diabetes	1 (Ref.)	–	–	1	–	–
Diabetes	2.59	9.80–8.42)	0.1	2.79	0.35–25.98	0.3

*OR* odds ratio, *CI* confidence interval, *AKI* acute kidney injury, *BMI* body mass index

In multivariable logistic regression models, preoperative CysC (OR: 9.3, CI: 1.8–67.8) and RN (OR: 8.3, CI: 2.1–38.7) were independent predictors for a reduced eGFR < 60 ml/min/1.73m^2^ at follow-up (both *p* ≤ 0.01). Conversely, postoperative CysC was not associated with an eGFR < 60 ml/min/1.73m^2^ at follow-up (*p* = 0.3). Additionally, when preoperative creatinine was used and adjusted for the same covariates, creatinine did not achieve independent predictor status for an eGFR < 60 ml/min/1.73m^2^ at follow-up (*p* = 0.2).

We further validated these observations in multivariable linear regression models (Table 3). Here, preoperative CysC (OR: − 5.8, CI: − 9.7–(− 2.03), *p* < 0.01) was also independently influencing the patients’ eGFR at follow-up. This means that a preoperative increase of the CysC level of 0.1 mg/dl was associated with an eGFR decline of 5.8 ml/min/1.73m^2^ at follow-up.

**Table 3:** Linear regression models predicting estimated glomerular filtration rate (eGFR) ml/min/1.73m^2^ in follow-up, after either partial (PN) or radical nephrectomy (RN) for renal cell carcinoma

	Univariable			Multivariable		
	Coefficient	95% CI	*p* value	Coefficient	95% CI	*p* value
Preoperative cystatin c	− 7.60	− 9.79–(− 5.40)	< 0.001	− 5.84	− 9.65–(− 2.03)	< 0.01
Postoperative cystatin c	− 3.69	− 4.79–(− 2.60)	< 0.001	− 0.02	− 2.13–2.08	1
Age at diagnosis	− 0.51	− 0.90–(− 0.13)	< 0.01	− 0.36	− 0.74–0.03	0.07
Tumor size	− 3.36	− 4.93–(− 1.80)	< 0.001	− 0.33	− 2.26–1.61	0.7
No postoperative AKI	1 (Ref)	–	–	1	–	–
Postoperative AKI	− 17.09	− 25.27–(− 8.91)	< 0.001	− 10.62	− 21.56–0.31	0.056
PN	1 (Ref)	–	–	1	–	–
RN	− 25.78	− 33.41–(− 18.15)	< 0.001	− 12.99	− 24.49–1.49	0.03
BMI	− 0.84	− 1.67–(− 0.01)	0.048	− 0.17	− 1.24–0.91	0.8
Preoperative diabetes	− 6.92	− 19.10–5.25	0.3	3.21	− 10.90–17.31	0.6
Preoperative hypertonia	− 5.55	− 14.92–3.83	0.2	− 1.48	− 10.60–7.64	0.7

*OR* odds ratio, *CI* confidence interval, *AKI* acute kidney injury, *BMI* body mass index

### Comparison between postoperative cystatin C vs. creatinine

**Fig. 1:**
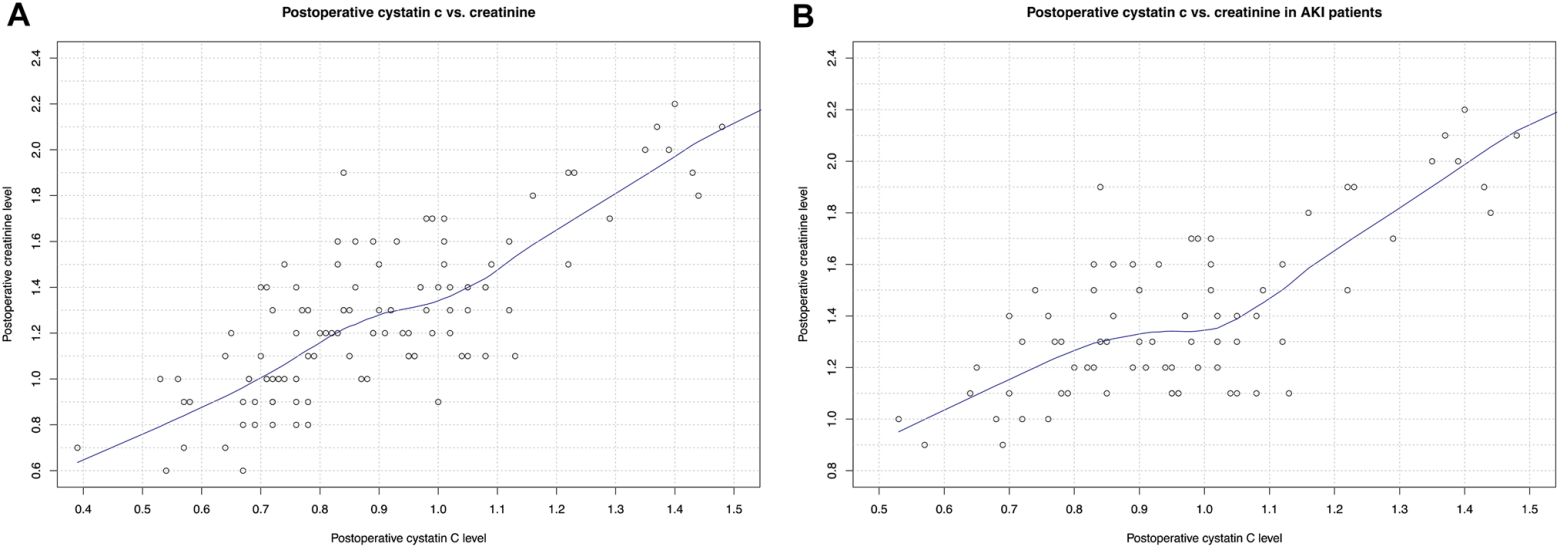
Loess plots depicting the relationship between postoperative cystatin c level and postoperative creatinine level in **A** all examined patients and in **B** patients with acute kidney injury (AKI) after kidney surgery for renal cell carcinoma

**Fig. 2:**
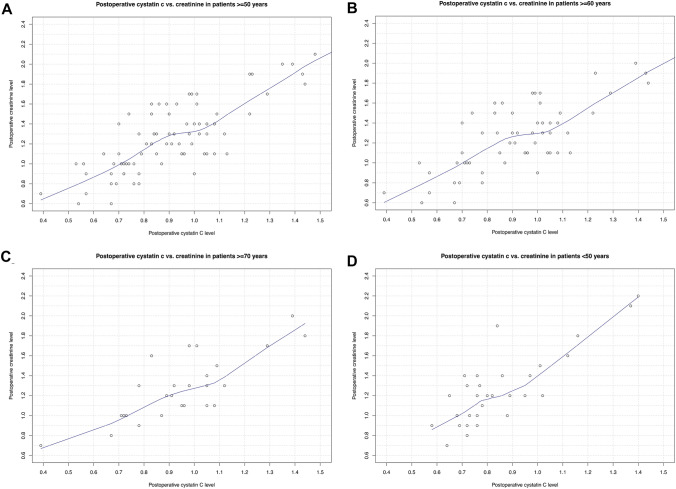
Loess plots depicting the relationship between postoperative cystatin c level and postoperative creatinine level in **A** patients aged ≥ 50 years in **B** patients aged ≥ 60 years **C** patients aged ≥ 70 years and **D** patients aged < 50 years after kidney surgery for renal cell carcinoma

To further investigate the performance of postoperative CysC in surgically treated patients, we plotted postoperative CysC values vs. creatinine values in all examined patients (Fig. 1A). Here, we observed a linear relationship between postoperative cystatin C and postoperative creatinine. The exception consisted of the range of postoperative CysC values between 0.9–1.0 mg/l. Here, a plateau at a postoperative creatinine range of approximately 1.2–1.3 mg/dl was observed. We further validated this observation in postoperative AKI patients (Fig. 1B). Moreover, we validated this observation in different age subgroups (≥ 50, ≥ 60, ≥ 70, < 50 years, Fig. 2). All stratification yielded virtually same observations.

## Discussion

Several studies suggested CysC as a better marker for kidney function estimations than creatinine, based on CysC’s molecular structure and its physiology regarding filtration and reabsorption [14, 27]. However, these assumptions have not been sufficiently validated in contemporary surgically treated RCC cohorts. We hypothesized that CysC may play a crucial role in outcome measurements of kidney function after surgical treatment with either PN or RN for RCC patients. Against this backdrop, our study holds several noteworthy findings.

First, we found that patients with postoperative elevated CysC after RCC surgery were older (61 vs. 59 years), underwent more frequently RN (48.6 vs. 16.7%) and presented larger tumor sizes (4.6 vs 3.4 cm), than patients with normal postoperative CysC level. Moreover, expectedly, patients with elevated postoperative CysC had a lower preoperative eGFR, and higher creatinine and CysC values, than patients with normal postoperative CysC. Additionally, patients with postoperative elevated CysC level more frequently exhibited diabetes (20 vs. 6%). These observations are not surprising, since they validate preoperative selection criteria for patients who are candidates for either PN or RN, as already observed in previous studies [28]. Here especially patients with higher age and more comorbidities underwent more frequently RN. In consequence, due to preoperative comorbidity profile and non-nephron sparing RN, these patients were at highest risk for postoperative elevated CysC levels, despite the fact that AKI rates between both groups did not differ. Taken together, further efforts should be made to provide nephron-sparing surgery to all patients, whenever feasible, to avoid postoperative kidney function impairment.

However, it is also particularly important that approximately 30% of all patients with elevated postoperative CysC were not classified as AKI, according to traditional criteria [29]. Therefore, clinicians should be aware of patients with risk profile for kidney function impairment, which may be underestimated by usual creatinine-based AKI definitions, when cystatin C tests in clinical practice are not routinely performed. These risk factors may be mainly the surgical approach, diabetes and age, as also already investigated in previous publications [11, 30].

Second, we also made important observation regarding cystatin C as a predictor for kidney function impairment at follow-up. Specifically, we observed in multivariable logistic regression models that preoperative cystatin C was an independent predictor of kidney function impairment in follow-up. Conversely, postoperative cystatin C level was not found to be such a predictor after adjusting for the above-mentioned risk factors of age, surgical approach and diabetes among others. Consequently, it is possible that a postoperative increase in the CysC level (measured on the first postoperative day) only reflects a physiological reaction on the performed surgery and is measured too early to predict valid long-term kidney function estimates. Conversely, the preoperative CysC level may better reflect the cumulative overall lifestyle and comorbidity-related kidney function and its potential to be of high risk for post-surgical impairment. These assumptions are also emphasized by the observations by Alesawi et al., where already five minutes after unclamping the kidney vessels an increase of the CysC level was observed [16].

Moreover, our observations were further validated in linear regression models, where an increase of the preoperative CysC blood level of 0.1 mg/l was independently associated with an eGFR reduction of − 5.8 ml/min/1.73m^2^ in the follow-up. To the best of our knowledge, we are the first to observe this important relationship of CysC in a surgically treated cohort of RCC patients. In consequence, our data cannot be directly compared to previous studies. However, in a previous study investigating the effect of CysC in a donor nephrectomy cohort for living-donor kidney transplantation, similar observations have been also reported. Here, cystatin C concentration was also independently associated with the occurrence of CKD in follow-up [31]. Nonetheless, important differences exist between donor nephrectomy and RCC nephrectomy patients regarding baseline characteristics, such as age at surgery (44 vs. 60 years) or comorbidity profiles (for example diabetes proportions (0.7 vs. 13.3%). In consequence, our findings provide an important and robust contribution for a specific RCC cohort regarding CysC as a predictor for kidney function impairment. Therefore, preoperative CysC levels can be used to estimate the extent of the kidney impairment after RCC surgery. The occurrence of CKD, as well as AKI is frequent in the cohort of patients undergoing RCC surgery. In consequence, a nephrology referral should be considered to address the increased risk for cardiovascular mortality, CKD progression and CKD-related comorbidities in these patients, and especially in those with elevated preoperative levels of CysC.

Third, we also made important observation regarding postoperative CysC level as a marker for kidney function impairment. Specifically, we observed that regardless of rising postoperative CysC level in the range of approximately 0.9–1.0 mg/l, the postoperative creatinine level remained at a plateau of 1.3 mg/dl. However, for postoperative CysC below 0.9 mg/l and above 1.0 mg/l a linear correlation between postoperative cystatin C and creatinine was observed. The same observations were further made in subgroup analyses of AKI patients and different age groups. These observations are particularly noteworthy, since in a postoperative setting kidney function impairment might not be detected in the range around 1.3 mg/dl creatinine value. In consequence, CysC might be a better marker for postoperative kidney function measurement. Moreover, a previous study by Roberts et al. showed that kidney function may be overestimated in older patients, when creatinine-based estimates are used [19]. However, in the current study, our observations suggest that CysC results in the same outcomes in elderly patients as in younger patients.

Our study has several limitations and needs to be interpreted in its retrospective, single-institution design. Some of our observations and findings may be limited by sample size, especially in subgroup analyses addressing elderly patients. Moreover, we relied on the MDRD formula for eGFR estimates, which may underestimate its values, relative to other formulas. Also, patients’ medication may have impacted eGFR estimates. Finally, only approximately the half of all the surgical treated patients of the current study participated in the follow-up, which may bias follow-up results and predictions. Follow-up was not obtained at standardized time points after RCC surgery. In consequence, all of the above findings need to be ideally further validated in RCC cohorts with bigger sample sizes.

Taken together, our results suggest that baseline patient characteristics such as age, as well as the comorbidity profile (especially diabetes) and the surgical approach identify patients with high risk of postoperative elevated CysC level and consecutive renal function impairment. Moreover, elevated CysC detected approximately 30% of patients with reduced kidney function, which may not have been identified through traditional creatinine-based AKI definitions. Second, we also observed that the preoperative CysC level was a predictor for long-term kidney function impairment and CKD prediction, while postoperative CysC level was not. However, the postoperative CysC level seems to be more sufficient to uncover early postoperative kidney function impairment than creatine values, especially in a creatinine range around 1.3 mg/dl.

## Data Availability

All datasets generated for this study are included in the manuscript.

## Code availability

On request used software R statistics (version 3.6.1).
